# Effect of the needle-free “intra dermal application of liquids” vaccination on the welfare of pregnant sows

**DOI:** 10.1186/s40813-017-0056-3

**Published:** 2017-04-04

**Authors:** Déborah Temple, Damián Escribano, Marta Jiménez, Eva Mainau, José J. Cerón, Xavier Manteca

**Affiliations:** 1 School of Veterinary Medicine, Universitat Autònoma de Barcelona, 08193 Bellaterra, Spain; 2 School of Veterinary Medicine, University of Murcia, 30100 Murcia, Spain; 3 MSD Animal Health, 37008 Carbajosa de la Sagrada, Salamanca Spain

**Keywords:** Intradermal vaccination, Sows, Behaviour, Welfare

## Abstract

**Background:**

In commercial pig production, sows are often vaccinated several times per gestation period, resulting in reduced welfare. This preliminary experiment investigated whether the needle-free IDAL vaccinator improves welfare through reduction of stress markers, improvement of behavioural and health parameters compared to traditional needle-syringe method.

**Results:**

Two treatments (IDAL and NEEDLE) in 6 replicate pens of gestating sows (15 sows per pen) were evaluated using Porcilis® PRRS. The frequency of sows exhibiting an acute fear (or pain) response at the time of injection was significantly lower in the IDAL sows for the four indicators studied (high pitch vocalizations, IDAL = 15.4% vs. NEEDLE = 95.6%, *χ*2 = 56, *P* < 0.0001; retreat attempts, IDAL = 2.6% vs. NEEDLE = 56.5%, *χ*2 = 28, *P* < 0.0001; turning back, IDAL = 5.1% vs. NEEDLE = 69.6%, *χ*2 = 36, *P* < 0.0001; change in behaviour, IDAL = 18% vs. NEEDLE = 95.6%, *χ*2 = 53, *P* < 0.001). Sows in the NEEDLE vaccination group had a decreased (*P* = 0.03) activity the day after vaccination compared to IDAL sows. No significant difference was observed for the other active behaviours and resting postures. Fearful reaction towards the assessor significantly (*χ*2 = 12, *P* = 0.001) increased in NEEDLE sows compared to IDAL sows the day after vaccination. At 48 h post-vaccination, IDAL sows tended to have lower blood C-reactive protein levels (IDAL = 21.3 μg/mL vs. NEEDLE = 35.8 μg/mL, *P* = 0.06) compared to NEEDLE sows. Blood Haptoglobin levels did not differ significantly between treatments 48 h post-vaccination. Chromogranin A tended to show a lower increase after the IDAL treatment, whereas salivary alpha-amylase and salivary cortisol did not differ between treatments when measured 25 min post-vaccination.

**Conclusions:**

These preliminary results support that needle-free intradermal vaccination is a promising strategy to reduce fear and pain reaction of gestating sows during vaccination.

## Background

In commercial pig production, sows are often vaccinated several times per gestation period. Vaccination is generally necessary to improve the health of the animals through prevention of diseases. However, vaccination can cause discomfort because of the systemic reaction and pain due to the local inflammation at the injection site. Among other husbandry tasks undertaken by stock people, vaccination is reported as a negative event for animals that can result in acute and chronic fear due to the painful procedure [1]. A variable degree of pain may be caused depending on the nature of the injection. Needle-syringe is the most common method for vaccination of pigs. Needle-syringe method has been associated with needle-site lesions that are the result of broken needles or bacterial contamination [2]. Needle-free technology has been developed to increase vaccine efficacy, safety, or compliance and potentially to minimize animal stress. According to several studies, needle-free devices offer advantages including elimination of broken needles, consistent vaccine delivery, lower vaccine volume and greater antigen dispersion, elimination of accidental worker needle sticks and elimination of needle disposal [2, 3]. In humans, needle-free method caused less pain and stress at the time of vaccination [4, 5]. By analogy, a similar effect should be expected in animals. Göller et al. [6] studied the effect of intradermal vaccination on the behaviour of suckling piglets. To the authors’ knowledge, the impact of needle-free injections on the behaviour and welfare in pigs has not been further investigated or reported. Needle-free devices differ on the power source: spring-powered, battery-powered and compressed-gas-powered [2]. This study will focus on the needle-free injector named “Intra Dermal Application of Liquids” or IDAL (MSD Animal Health, Boxmeer, The Netherlands), which is a battery powered injector.

Both the Hypothalamic-Pituitary axis (HPA) and the sympathetic adrenomedullary (SAM) system can be activated in response to vaccination. Handling, restraint, fear and pain are some emotional and physical stressors related to vaccination. Biomarkers react differently to different types of stressors and the use of a panel of stress markers is essential to quantify the stress response to a given situation. Saliva is considered an ideal sample for evaluating stress conditions in pigs, by using stress biomarkers [7, 8]. In comparison with blood sampling, saliva sampling is generally considered to be a non-invasive and stress-free methodology [9]. The HPA axis and the SAM system play a key role in the stress response [10]. Salivary cortisol indicates activity of the HPA axis in response to different stressors in pigs [9]. Salivary α-amylase (sAA) and Chromogranin-A (CgA) have been shown to be reliable alternatives to catecholamines for monitoring SAM activity, which constitutes the initial and “fast” response to stress. Fuentes et al. [11] observed that sAA increases after a model of stress induction in pigs. CgA has been used as a sensitive and reliable indicator to monitor activity of the sympathetic nervous system in humans [12, 13]. Recently, CgA has been reported as an acute stress indicator in pigs [14]. Thereafter, salivary cortisol, sAA and CgA will be used as biomarkers of the stress reaction of sows to vaccination. Other biomarkers, the acute phase proteins (APPs), are a group of blood proteins that change in concentration in animals subjected to external or internal challenges, such as infection, inflammation, surgical trauma and stress [15]. Vaccination may produce an increase of APPs such as the C-reactive protein (CRP) and the Haptoglobin (Hp) through the activation of the acute phase response, the non-specific innate immune response [16]. Even though it has been hypothesized that APPs secretion can be induced by metabolites released through the SAM and/or HPA pathways [15], recent studies do not support the use of Hp and CRP as indicators of acute emotional stress [8, 17]. CRP and Hp will therefore be used in the present study as more specific biomarkers of the inflammation induced by vaccination.

In summary, very little is known about the welfare benefits of using needle-free vaccination in pigs. This study presents the behavioural and physiological response of sows to needle-free IDAL vaccination compared to the needle-syringe method. Health indicators such as skin reaction at the site of injection and rectal temperature were also monitored.

## Methods

### Animals, housing and experimental procedure

A total of 90 pregnant sows (Landrace x Duroc) were included in the study. Sows were allocated to 6 identical pens on a commercial farm 28 days after service. Groups were static and the animals were managed following the usual routine of the farm. All sows were multiparous at 48–80 days of gestation at the beginning of the study. Sows were housed in groups of 15 animals. Pens (8 × 5.5 m, 2.9 m^2^/sow) had concrete floors and solid concrete partitions which prevented any physical contact with sows in neighbouring pens. Sows were fed twice a day at 8 am and 6 pm from partial stalls and had *ad libitum* access to water.

Sows were vaccinated either by needle-free IDAL method (IDAL) or conventional needle-syringe (NEEDLE) using the vaccine Porcilis®PRRS (MLV European strain, MSD Animal Health). The volume of vaccine administered was 0.2 ml in the IDAL group and 2 ml in the NEEDLE group. Vaccinations were performed by two trained technicians, one for each vaccination method. Treatments were alternated from one pen to another starting vaccinations at 11 am and finishing at 6 pm.

### Behavioural measures

Behavioural indicators of fear or pain at the time of injection (high pitch vocalizations, retreat attempts, turning back and changes in activity) were recorded at individual level and scored as present or absent.

The reactivity of each individual sow towards a person present in the pen was evaluated using the fear to human test validated by the Welfare Quality® (WQ®) for sows [18] the day before and +24 h after the vaccination. The scoring scale ranged from 0 (the sow allows the observer to touch her between the ears without any withdrawal response, or the sow withdraws when it is attempted to touch her but then approaches) to 2 (extreme withdrawal response).

Social behaviour and general activity were recorded the day before and after the vaccination by means of scan samplings adapted from the methodology proposed by the WQ® protocol for sows on farm [18]. Pigs were scored as either active or inactive. The behaviours recorded from active sows were as follows: positive social behaviour, negative social behaviour, exploratory behaviour, drinking, and other (observing the observer, walking, gazing, etc.) (Table 1).

**Table 1:** Ethogram for direct observations (scan sampling) at 24 h pre- and post- vaccination (adapted from the WQ protocol for sows [18])

Behavioural Category	Definition
Active behaviour	
Social negative behaviour	Aggressive behaviour, including biting or any social behaviour with a fearful or aggressive response from the disturbed animal
Positive social behaviour	Sniffing, nosing, licking and moving gently away from the animal without an aggressive or flight reaction from this individual
Drinking	Mouth on water trough
Exploring	Sniffing, nosing, licking or chewing all features of the pen or paddock and manipulation of enrichment material
Other active behaviours	All other active behaviours (air sniffing, gazing, walking etc.)
Non-Active behaviour	
Resting	Sleeping (lying down with the eyes shut)

Observations took place the day before (day −1) and day after (day +1) vaccination, with three observation blocks per day for each pen (from 10 am to 4 pm). Observations took place outside of the sows’ feeding time (7 am and 5 pm). Within an observation block, each pen was observed 5 times with an interval of 2.5 min between two scans. The number of sows engaged in each social behaviour category (social negative and social positive) and four general active behaviours (drinking, exploration and other) were recorded. Social behaviour, drinking, exploration and “other” active behaviour were expressed in proportion of the total number of active pigs. The percentage of active pigs was expressed in proportion to the total number of observations (active + resting animals). Resting posture was recorded at the same time, by means of the same scan sampling and counting the number of sows lying and sitting. Moreover, when pigs were lying, a determination was made as to whether they were lying ventrally or laterally.

All observations were carried out by a single observer previously trained to apply the WQ® protocol for sows on farm [18].

### Sampling of physiological parameters

From the total of 90 sows, 54 were randomly selected and marked with a spray before beginning the saliva sampling. Saliva was adequately obtained from 44 sows. Salivary samples for sAA, CgA and salivary cortisol determination were obtained by introducing a small sponge in the pigs’ mouth for at least 30 s with the help of a metal rod. The sponges were placed in a plastic tube and were centrifuged at 3000 g for 10 min at 4 °C. Saliva samples were aliquoted and stored at −22 °C until analysis. Salivary cortisol level was analysed using an automated chemiluminescent immunoassay validated for pigs [19]. Salivary CgA was determined by time-resolved immunofluorometric assay described by Escribano et al. [14]. sAA activity was determined by kinetic spectrophotometric assay validated by Fuentes et al. [11]. Before the trial, sows received 7 days adaptation to the saliva sampling. Previously to the salivary sampling, all sows were habituated and conditioned individually to the small sponge. Conditioning was necessary to avoid possible initial fear reaction to the saliva sampling. Sponges were soaked in apple juice and classical conditioning was considered sufficient when the sow grabbed the small sponge (without apple juice) without showing any fear reaction. Two saliva samples were obtained from each sow, one immediately before vaccination (T0) and a second one 25 min (T1) after vaccination.

From the sows sampled for salivary parameters, 30 sows were randomly selected and marked with a spray at 48 h post-vaccination for APP determination: Hp and CRP. Blood was extracted from the jugular vein, centrifuged at 2000 g for 5 min and kept at −80 °C until analysis. Haemolysis was observed in two blood samples that were finally discarded. Porcine CRP and Hp were measured by an automated biochemistry analyser (Olympus AU2700, Olympus Diagnostica GmbH, Hamburg, Germany) previously validated in pigs [20, 21].

### Health measures

Health measures were assessed on the 44 sows included in the sampling of physiological parameters.

Rectal temperature was assessed with a thermometer on day −1, +1 (+28 h), +2 (+52 h) and +7 of the vaccination.

Skin reaction at the point of vaccine injection was assessed on day 0, +1 (+28 h), +2 (+52 h), +7 and +28 of the vaccination. The skin reaction was defined as a reddish and hard local reaction at the site of the injection and its diameter was measured in centimetres.

### Statistical analysis

Behavioural data from scan samplings were analysed by means of non-parametric GEE models using the GENMOD procedure. The pen was the statistical unit for all behavioural data obtained by scan sampling. The proportion of sows showing high pitch vocalizations, retreat attempts, turning back, changes in activity at the time of vaccine injection and an increased panic response in the fear to human test following each vaccination treatment was compared between treatments using a Chi-square test.

Physiological data and rectal temperature were tested using a general linear mixed model PROC MIXED in SAS. Cortisol, CgA, sAA and CRP were log transformed. The model included the main effect of the treatment (2 levels). For salivary parameters and rectal temperature, start level on day −1 was included as covariate.

The proportion of sows showing a skin reaction at the site of injection was compared between treatments using a Chi-square test.

## Results

### Behavioural data

Table 2 summarizes the effect of IDAL vaccination on social behaviour, general activity and resting patterns. The occurrence of social behaviour was not affected by the vaccination method. Sows from the NEEDLE treatment had decreased (*P* = 0.0309) activity the day after vaccination compared to the IDAL sows. No significant difference was observed for the other resting patterns.

**Table 2:** Mean occurrence and SD of active behaviours and resting postures recorded during the scan sampling of sows from the IDAL and NEEDLE groups

24 h Before Vaccination			
Social behaviour and other active behaviours (%*)*	IDAL	NEEDLE	*P*
Social negative	1.1 ± 1.52	3.1 ± 3.19	0.0582
Social positive	3.1 ± 4.02	5.6 ± 5.13	0.3307
Drinking	2.1 ± 2.89	4.0 ± 3.87	0.2343
Exploration	21.8 ± 12.98	30.2 ± 8.04	0.2124
Active total	53.1 ± 28.83	55.6 ± 24.89	0.8212
Resting posture (%)			
Lying total	55.9 ± 29.36	54.1 ± 26.18	0.9021
Lying ventrally	90.0 ± 15.41	93.4 ± 8.45	0.3979
Sitting	0.9 ± 2.14	0.6 ± 1.39	0.6880
24 h Post-Vaccination			
Social behaviour and other active behaviours (%)			
Social negative	4.1 ± 2.90	5.1 ± 3.93	0.4919
Social positive	2.4 ± 2.44	2.3 ± 2.76	0.9277
Drinking	4.7 ± 2.65	3.2 ± 4.82	0.4757
Exploration	18.0 ± 9.64	21.6 ± 14.69	0.6060
Active total	45.6 ± 17.52	29.1 ± 19.11	0.0309
Resting posture (%)			
Lying total	67.2 ± 17.07	78.0 ± 19.91	0.8321
Lying ventrally	72.0 ± 15.99	65.1 ± 12.10	0.2202
Sitting	1.4 ± 2.41	1.1 ± 2.10	0.1901

The frequency of sows exhibiting an acute fear (or pain) response at the time of injection was significantly lower in the IDAL sows for the four indicators studied (high pitch vocalizations, IDAL = 15.4% vs. NEEDLE = 95.6%, *χ*2 = 56, *P* < 0.0001; retreat attempts, IDAL = 2.6% vs. NEEDLE = 56.5%, *χ*2 = 28, *P* < 0.0001; turning back, IDAL = 5.1% vs. NEEDLE = 69.6%, *χ*2 = 36, *P* < 0.0001; change in behaviour, IDAL = 18% vs. NEEDLE = 95.6%, *χ*2 = 53, *P* < 0.001).

The proportion of sows showing a fearful reaction towards the assessor significantly (*χ*2 = 12, *P* = 0.0006) increased in the NEEDLE group compared to IDAL sows the day after vaccination. Indeed, 33% of sows in the NEEDLE group that did not show any sign of fear before the vaccination (score 0) exhibited a total withdrawal (score 2) from the observer during the fear to human test the day after vaccination, compared to 3% in the IDAL group.

### Physiological data

A large inter-individual variability in salivary biomarker concentration was measured the day before and after vaccination (Table 3), especially for salivary sAA which presented high coefficients of variation (CV), including the day before the vaccination. CgA levels tended to be higher in sows vaccinated by the needle-syringe. Salivary α-amylase and cortisol did not differ between treatments.

**Table 3:** Means, SD and CV of salivary cortisol, Alpha-Amylase and, Chromogranin-A for the IDAL and NEEDLE group immediately before and after (+25 min) vaccination

		Mean ± SD (CV)		Effects (*P*)	
		IDAL	NEEDLE	Treatment	Baseline (before)
Cortisol (μg/dL)	Before	0.47 ± 0.18 (38%)	0.45 ± 0.22 (51%)		
	After	0.50 ± 0.21 (42%)	0.42 ± 0.11 (27%)	0.5771	0.0062
α-amylase (UI/L)	Before	4908 ± 7797 (159%)	5053 ± 6501 (128%)		
	After	3381 ± 5684 (168%)	2877 ± 3167 (110%)	0.7684	0.0383
Chromogranin-A	Before	0.23 ± 0.23 (100%)	0.21 ± 0.15 (71%)		
(μg/mL)	After	0.43 ± 0.44 (102%)	0.88 ± 1.01 (126%)	0.0743	0.0001

### Health data

Prevalence of sows with skin alterations at the site of injection is shown in Table 4. From 44 sows evaluated, 21 were from the IDAL group and 23 from the NEEDLE group. However, two IDAL sows could not be evaluated properly for practical reasons (dark skin colour). A reddish skin reaction of 0.5 cm diameter was observed in 47% of IDAL sows at +28 h while 9% of NEEDLE sows presented a skin reaction at the site of injection. At + 28 days, no IDAL sows presented any sign of skin alteration whereas 26% of sows from the NEEDLE vaccination group presented 3 cm diameter abscesses at the site of injection.

**Table 4:** Mean percentage of sows from the IDAL and NEEDLE groups with a skin alteration at the injection site on day +1 (+28 h), +2 (+52 h), +7 and +28 post-vaccination

	Day + 1	Day + 2	Day + 7	Day + 28
IDAL	47% (9/19)	53% (10/19)	5% (1/19)	0% (0/19)
NEEDLE	9% (2/23)	22% (5/23)	22% (5/23)	26% (6/23)
*P*	0.0215	0.0428	0.3136	0.0351

Rectal temperature was not significantly different between treatments (Table 5).

**Table 5:** Mean rectal temperature and SD of sows the day before the vaccination and day +1 (+28 h), +2 (+52 h) and day +7 post vaccination

	Day −1 (baseline)	Day + 1	Day + 2	Day + 7
IDAL	37.3 ± 0.73 °C	37.2 ± 0.75 °C	36.8 ± 1.12 °C	37.7 ± 0.54 °C
NEEDLE	37.7 ± 0.62 °C	37.6 ± 0.38 °C	36.9 ± 0.87 °C	37.5 ± 0.85 °C
*P*	ns	ns	ns	ns

*n*s not significant (*P* >0.05)

## Discussion

The present study evaluated the effect of the needle-free IDAL vaccination method on the welfare of gestating sows through behavioural, physiological and health parameters.

Behaviour is a sensitive indicator of the animal’s perception of environmental changes. Variations in behavioural patterns often represent the first level of response of an animal to an aversive or stressful situation. Fear reactions are the most immediate responses that animals show to potentially dangerous stimuli in the environment [22]. The frequency of high pitch vocalizations, retreat attempts and turning back were significantly higher in sows vaccinated with the needle-syringe method. All these behaviours have been suggested as indicators of pain and anxiety [1, 23] in pigs. Thereafter, the needle-free IDAL vaccination method prevents sows developing acute fear and pain at the time of injection compared to conventional needle-syringe vaccination. The vaccination method appeared to be a strong determinant of the fear reaction that the sows show in the fear to human test the day after the vaccination. One third of the sows vaccinated with the needle-syringe exhibited a total withdrawal from the observer 24 h after the vaccination while being curious and friendly the day before the vaccination. Such change in the fearful reaction was not observed in sows vaccinated with IDAL. Vaccination method appeared to modify animal’s perceptions of humans the day after vaccination. Long term effect of the vaccination method on fear reaction of the animal, productivity, and reproduction is unknown but deserves further investigation.

General activity was significantly affected by the vaccination method; sows vaccinated with the needle-syringe method reduced their activity the day after vaccination compared to the IDAL sows. Other indicators of resting pattern such as body postures were not altered by the vaccination and rectal temperatures did not vary across treatments. Difference in general activity should therefore not be related to a febrile response. Instead, rest may increase in sows vaccinated with the needle-syringe method the day after vaccination as part of the recovery process from the fear and pain reaction during the vaccine application. Similarly, Göller et al. [6] reported a greater activity in suckling piglets the day after intradermal vaccination compared with the intramuscular group. Intradermal piglets also presented an increased suckling behaviour which was associated to a reduced degree of stress [6].

While the physiological changes of pigs exposed to social and physical stressors such as mixing or fasting has been demonstrated [24], no study reports the effect of vaccination method on stress biomarkers. Fear is associated with physiological stress. Given the strong difference in acute fear reaction to the vaccination method, physiological differences between vaccination treatments were expected. In the present study, salivary CgA tended to increase in sows vaccinated with the needle-syringe method. CgA is considered as a marker of acute stress and Escribano et al. [14, 17] reported an increase in CgA levels in pigs during restraint and after isolation and regrouping. Gallina et al. [13] showed a high correlation between CgA and cardiovascular parameters during high-intensity exercise. In a recent study CgA was increased after feed deprivation indicating a possible influence of metabolic stress on CgA [24]. Increased concentrations of CgA in the sows vaccinated with the needle-syringe method may either reflect an acute stress response related to the management procedure and increased cardiovascular activity or to a possible metabolic change produced by the acute pain reaction to the injection. In the present study, the tendency of salivary CgA to show a lower increase after the IDAL treatment was not detected in sAA.. This may be explained by different regulation pathways of both biomarkers as also commented by Gallina et al. [13] when studying the effect high-intensity exercise. Mean levels of sAA before the vaccination were comparable to high activity levels reported by earlier research that used the same techniques [11] and a very high variability between animals was observed. Many factors can influence sAA production such as food intake, exercise, or circadian rhythm [25]. The sows appeared to be so much conditioned to the saliva sampling that their level of arousal increased when just seeing the little sponge. This anticipatory behaviour toward sampling may explain the high sAA levels before the vaccination. This may have influenced CgA levels as well, reducing differences between treatments. No significant changes in salivary cortisol were observed in the present study. This may indicate that HPA axis has not been activated by the vaccination procedure. Jaskulke and Manteuffel [26] report a high capacity of adaptation of the pig HPA axis to stressful situations. Based on our results, salivary cortisol may not be sensitive enough to measure acute fear reaction after vaccination.

CRP and Hp are two APPs which concentrations change considerably upon inflammatory stimulus [27]. Concentration of CRP and Hp are related to the severity of underlying diseases [28]. In apparently healthy pigs, Pallarés et al. [29] found serum levels of Hp and CRP significantly higher in animals with lesions at slaughter than those without lesions. Destexhe et al. [30] showed that CRP can be used as a biomarker of acute inflammation one day after vaccine administration. In the present study, levels of CRP tended to be higher in sows vaccinated with the needle-syringe method with a difference of 1.7 times the value of IDAL sows. Carpintero et al. [31] reported up to ten-fold increases during acute processes. Therefore, the stress and inflammation caused by needle-syringe compared to IDAL vaccination may result in higher levels of CRP after 48 h. Differences in CRP levels may be due to tissue damage caused by the injection or physical and psychological stress linked to the vaccination procedure. Still, no strong affirmation can be done as for practical constraints APPs basal levels could not be evaluated before the vaccination. Around 50% of the sows presented a skin reaction at the site of injection 28 h and 52 h following the IDAL vaccination. This reaction was described as a reddish inflammation of 0.5 cm diameter. The skin reaction was not more visible 28 days post-vaccination in IDAL sows. On the contrary, 9% of sows vaccinated with the needle-syringe method showed a skin reaction after 28 h but this percentage increased at 26% of affected animals at 28 days. This latter skin reaction was described as an abscess, sometimes with opened lesion, of 3 cm diameter. Abscesses may result from injections when using contaminated needles [32].

## Conclusions

In commercial pig production, sows are often vaccinated several times per gestation period which can result in a reduced welfare. Stress markers and behavioural parameters show that needle-free “Intra Dermal Application of Liquids” (IDAL) vaccination reduces sow’s fear and pain reaction to the vaccination procedure compared to the needle-syringe vaccination method. The absence of any skin reaction at the site of injection 28 days post vaccination and lower CRP levels in IDAL sows compared to sows vaccinated with the needle-syringe show that IDAL vaccination may prevent the acute phase response and long term muscular damage associated to the injection. IDAL vaccination is therefore a very promising strategy to improve the welfare of gestating sows when vaccinated.

## Abbreviations

APPs: Acute phase proteins
CgA: Chromogranin_A
CRP: C-reactive protein
CV: Coefficient of variation
Hp: Haptoglobin
HPA: Hypothalamic-pituitary axis
IDAL: Intra dermal application of liquids
NEEDLE: Needle-syringe application
sAA: Salivary alpha-amylase
SAM: Sympathetic adrenomedullary
WQ: Welfare quality

## References

[1] Hemsworth PH, Coleman GJ (2010). Human-Livestock interactions.

[2] Chase CCL, Scanlon D, Garcia R, Milward F, Nation T (2008). Needle-free injection technology in swine: progress toward vaccine efficacy and pork quality. J Swine Health Prod.

[3] Willson P (2004). Needle free immunization. Pig Progress.

[4] Sarno MJ, Blasé E, Galindo N, Ramierez R, Schirmer CL, Trujillo-Juarez DF (2000). Clinical immunogenicity of measles, mumps and rubella vaccine delivered by the Injex jet injector: comparison with standard syringe injection. Ped Inf Dis J.

[5] Stout RR, Gutierrez MJ, Roffman M, Marcos J, Sanchez R, Macias M, Quiroz R, Taylor D, Mckenzie B, Restrepo E, Pinera I, Craig-Rodriguez A, Restrepo M, Turner P, Richardson R, Baizer L, Walker M, Walker E (2004). Subcutaneous injections with a single-use, pre-filled, disposable needle-free injection device or needle and syringe: comparative evaluation of efficacy and acceptability. Drug Delivery Tech.

[6] Göller M, Knöppel HP, Fiebig K, Kemper N. Intradermal vaccine application: effects on suckling behaviour. 24^th^ Int Pig Vet Soc Congr, Dublin. 2016; p. 625.

[7] Muneta Y, Yoshikawa T, Minagawa Y, Shibahara T, Maeda R, Omata Y (2010). Salivary IgA as a useful non-invasive marker for restraint stress in pigs. J Vet Med Sci.

[8] Soler L, Gutiérrez AM, Escribano D, Fuentes M, Cerón JJ (2013). Response of salivary haptoglobin and serum amyloid A to social isolation and short road transport stress in pigs. Res Vet Sci.

[9] Merlot E, Mounier AM, Prunier A (2011). Endocrine response of gilts to various common stressors: a comparison of indicators and methods of analysis. Physiol Behav.

[10] Koolhaas JM, Bartolomucci A, Buwalda B, de Boer SF, Flügge G, Korte SM (2011). Stress revisited: a critical evaluation of the stress concept. Neurosci Biobehav Rev.

[11] Fuentes M, Tecles F, Gutiérrez A, Otal J, Martínez-Subiela S, Cerón JJ (2011). Validation of an automated method for salivary alpha-amylase measurements in pigs (Sus scrofadomesticus) and its application as a stress biomarker. J Vet Diagn Invest.

[12] Nakane H, Asami O, Yamada Y, Harada T, Matsui N, Kanno T (1998). Salivary chromogranin A as an index of psychosomatic stress. Biomed Res.

[13] Gallina S, Di Mauro M, D’Amico MA, D’Angelo E, Sablone A, Di Fonso A (2011). Salivary chromogranin A, but not alfa-amylase, correlates with cardiovascular parameters during high-intensity exercise. Clin Endocrinol.

[14] Escribano D, Soler L, Gutiérrez AM, Martinez-Subiela S, Cerón JJ (2013). Measurement of chromogranin A in porcine saliva: validation of a time-resolved immunofluorometric assay and evaluation of its application as a marker of acute stress. Anim.

[15] Murata H, Shimada N, Yoshioka M (2004). Current research on acute phase proteins in veterinary diagnosis: an overview. Vet J.

[16] Gruys E, Toussaint MJM, Niewold TA, Koopmans SJ (2005). Acute phase reaction and acute phase proteins. J Zhejiang Univ Sci B.

[17] Escribano D, Gutiérrez AM, Tecles F, Cerón JJ (2015). Changes in saliva biomarkers of stress and immunity in domestic pigs exposed to a psychosocial stressor. Res Vet Sci.

[18] Welfare Quality® 2009. Welfare Quality® assessment protocols for pigs (sows and piglets, growing and finishing pigs). Welfare Quality® Consortium, Lelystad, the Netherlands.

[19] Escribano D, Fuentes-Rubio M, Cerón JJ (2012). Validation of an automated chemiluminescent immunoassay for salivary cortisol measurements in pigs. J Vet Diagn Invest.

[20] Kjelgaard-Hansen M, Martínez-Subiela S, Petersen HH, Jensen AL, Cerón JJ (2007). Evaluation and comparison of two immunoturbidimetric assays for the heterologous determination of porcine serum C-reactive protein. Vet J.

[21] Tecles F, Fuentes P, Martínez-Subiela S, Parra MD, Muñoz A, Cerón JJ (2007). Analytical validation of commercially available methods for acute phase proteins quantification in pigs. Res Vet Sci.

[22] Andersen IL, Berg S, Boe KE, Edwards SA (2006). Positive handling in late pregnancy and the consequences for maternal behaviour and production in sows. Appl Anim Behav Sci.

[23] Dalmau A, Velarde A, Forkman B (2009). Validation of measures of fear. Assessment of animal welfare measures for sows, piglets and fattening pigs. Welfare quality reports, N°10.

[24] Ott S, Soler L, Moons CP, Kashiha MA, Bahr C, Vandermeulen J, Janssens S, Gutiérrez AM, Escribano D, Cerón JJ, Berckmans D, Tuyttens FA, Niewold TA (2014). Different stressors elicit different responses in the salivary biomarkers cortisol, haptoglobin, and chromogranin A in pigs. Res Vet Sci.

[25] Nater UM, Rohleder N (2009). Salivary alpha-amylase as a non-invasive biomarker for the sympathetic nervous system: current state of research. Psychoneuroendocrinology.

[26] Jaskulke S, Manteuffel G (2011). No apparent effect of an experimental narrow confinement on heart activity and cortisol in domestic pigs. Anim.

[27] Cray C, Zaias J, Altman NH (2009). Acute phase response in animals: a review. Comp Med.

[28] Eckersall PD (2000). Acute phase proteins as markers of infection and inflammation: monitoring animal health, animal welfare and food safety. Ir Vet J.

[29] Pallarés FJ, Martínez-Subiela S, Seva J, Ramis G, Fuentes P, Bernabé A, Muñoz A, Cerón JJ (2008). Relationship between serum acute phase protein concentrations and lesions in finishing pigs. Vet J.

[30] Destexhe E, Prinsen MK, van Schöll I, Kuper CF, Garçon N, Veenstra S, Segal L (2013). Evaluation of C-reactive protein as an inflammatory biomarker in rabbits for vaccine nonclinical safety studies. J Pharmacol Toxicol Methods.

[31] Carpintero R, Piñeiro M, Andrés M, Iturralde M, Alava MA, Heegaard PMH, Jobert JL, Madec F, Lampreave F (2005). The concentration of apolipoprotein A-I decreases during experimentally induced acute processes in pigs. Infect Immun.

[32] Cameron R. Diseases of the skin. In: Straw B, Zimmerman J, D’Allaire S, Taylor D, editors. Diseases of swine. Oxford: Blackwell Publishing; 2006. p. 188.

